# Abdominal and Multifidus Muscle Morphology and Function, Trunk Clinical Tests, and Symmetry in Young Elite Archery Athletes

**DOI:** 10.3390/jcm14175974

**Published:** 2025-08-24

**Authors:** Gali Dar, Alon Yehiel, Kerith Aginsky, Yossi Blayer, Maya Calé-Benzoor

**Affiliations:** 1 Department of Physical Therapy, Faculty of Social Welfare & Health Sciences, University of Haifa, Haifa 3103301, Israel; alon.yehiel@gmail.com (A.Y.); mayac@wingate.org.il (M.C.-B.); 2 The Ribstein Sports Medicine and Research Center, Wingate Institute, Netanya 42902, Israel; keritha@wingate.org.il (K.A.); physiotherapy@wingate.org.il (Y.B.)

**Keywords:** archery, shooting sports, trunk stability, asymmetry, abdominal muscles, multifidus, back muscles, ultrasound, sport, strength

## Abstract

**Background/Objectives**: Archery is a technical sport involving repetitive and asymmetrical movements that requires trunk stability to enable good performance of the upper extremities. Being an asymmetrical sport, imbalances between sides might appear in the abdominal and back muscles. To assess trunk muscle function and symmetry in young competitive archers. **Methods**: Analyzing pre-season screening evaluation tests from medical files. This included an ultrasound examination of back and abdominal muscles (transverse abdominus and internal oblique) during rest and contraction and trunk muscle clinical strength tests. **Results**: Data on 15 elite archery athletes (mean age 17.2 (±2.7) years) were included. No athletes reported low back pain. No differences were found between the dominant and non-dominant sides in all outcome measurements (absolute thickness and percentage difference). Internal oblique muscle thickness during rest and contraction for the dominant side was higher in males compared with females (*p* < 0.05). The back muscles were more symmetrical than the abdominal muscles. **Conclusions**: Despite the asymmetrical functional demands of sport archery, young athletes displayed trunk muscle symmetry, particularly in their back muscles. While some variability in abdominal muscle asymmetry was observed, these differences were not statistically significant.

## 1. Introduction

Archery is a sport that is gaining popularity across various age groups. According to the Archery Trade Association, in 2014, approximately 21.6 million adults in the United States engaged in archery, representing 9.2% of the adult population. Participation included various forms of target archery, bowhunting, or both. These participation statistics include both recreational and professional athletes engaged in archery, highlighting the diverse range of individuals involved in the sport [1,2].

Archery is a technical sport where high-performance shooting is defined as the ability to shoot an arrow accurately at a specified target [3]. Archery involves a series of repetitive phases that follow a consistent sequence of movements, from drawing the arrow to releasing it. Athletes pull against the bowstring’s tension (ranging from 9 to 18 kg) while aligning their non-dominant hand with the target. Meanwhile, the dominant hand dynamically draws and releases the bowstring [4,5].

Archery is a highly isometric sport that requires strength and endurance in the upper body, arms, shoulder girdle, forearms, and fingers. Additionally, maintaining stability during lower limb movements is essential for body control [5,6,7].

From a biomechanical perspective, the repetitive and asymmetrical movements involved in drawing and releasing a bow are recognized as the primary cause of injuries in this sport, particularly affecting the upper extremities. The most frequently injured areas for archery athletes are the shoulders, followed by the elbow, and, to a lesser extent, the wrist [8]. The locations of the most common injuries vary depending on the type of bow used. For instance, Olympic archers often experience hypertrophy of the flexor tendons in their fingers, while those using compound bows are more susceptible to injuries in the shoulder area [4]. Although it is a non-contact sport, injuries can still occur, particularly soft tissue injuries affecting tendons, ligaments, and nerves [9].

Trunk muscles are important for upper limb movement and coordination and are activated isometrically prior to any upper extremity movement to maintain correct stance and balance [10]. The stability of the trunk during dynamic movements and static positions creates a strong base, enabling the upper extremities to generate movement, strength, good technique, speed, and coordination in various sports [11]. Trunk extensor muscles (erector spine and deep muscles) are constantly contracted to maintain posture against gravity. The abdominal muscles also contract isometrically to prevent extension of the spine and maintain trunk stability. The balance between the back and abdominal muscles is essential in preventing body sway and maintaining correct body posture and equilibrium [12]. Thus, during sport archery, trunk muscles are required for stability, enabling good performance of the upper extremities.

Because archery is an asymmetrical sport, asymmetries or imbalances between sides might appear in the abdominal and back muscles. The current study aims to assess trunk muscle function and symmetry among young competitive archery athletes. We hypothesized that asymmetry would be observed in the thickness of the abdominal and back muscles, even in young archery athletes, with the dominant side being thicker. This study will enhance the understanding of the physical aspects of archery and its demands, allowing for better preparation of the athlete participating in the sport.

## 2. Materials and Methods

### 2.1. Study Design

This was a retrospective study on data retrieved from the Ribstein Center for Sport Medicine Sciences and Research, Wingate Institute patient’s database. The study protocol was approved by the Institutional Review Board (#212024).

### 2.2. Data Source

Data were obtained from electronic medical files from the database of the Ribstein Sports Medicine and Research Center, which is the National Institute for Excellence in Sports in the State of Israel. This multidisciplinary center consists of physicians, orthopedists, physiotherapists, physiologists, nutritionists, and mental counsellors who work in full cooperation and supply professional support for athlete achievement and the Olympic and Paralympic teams of Israel.

The medical files of archery athletes were screened. The files contained demographic information (e.g., age and sex) and medical records (e.g., examinations, treatments, and medical conditions).

We summarized and analyzed examinations performed by physical therapists at the beginning of the season as pre-season screening evaluations. This included an ultrasound examination of the back and abdominal muscles and trunk muscle clinical strength tests.

All tests analyzed in this study were part of a series of screening assessments conducted before and during the archery season for the athletes. These screenings were performed by physical therapists from the Physical Therapy Clinic, who are specialized and have extensive experience in sports therapy. Th analysis also included any complaints or injuries recorded in the medical files.

### 2.3. Description of the Examinations

#### 2.3.1. Ultrasound Assessment

Ultrasound examinations were performed using a portable ultrasound device (Mindray M5, Shenzhen, Guangdong, China) with a 6 MHz convex transducer. All measurements were performed by an experienced physiotherapist who is qualified in ultrasound examinations.

Multifidus muscle assessment was performed while the athlete was in a prone position. The transducer was placed longitudinally and lateral to the spinous process and angled slightly medially until the L4/5 and L5/S1 zygapophyseal joint was identified. The split screen in B mode was used to assess muscle thickness at rest and during activation, utilizing a contralateral active straight leg extension. Multifidus muscle thickness was measured using the on-screen caliper, measured from the facet joint to the subcutaneous tissue. Each measurement was conducted twice, and the average of the trials was calculated for analysis. The procedure involved examining the multifidus muscles bilaterally. Specifically, the right multifidus muscle was assessed during left leg extension, while evaluation of the left multifidus was conducted during right leg extension [13].

**Figure 1:**
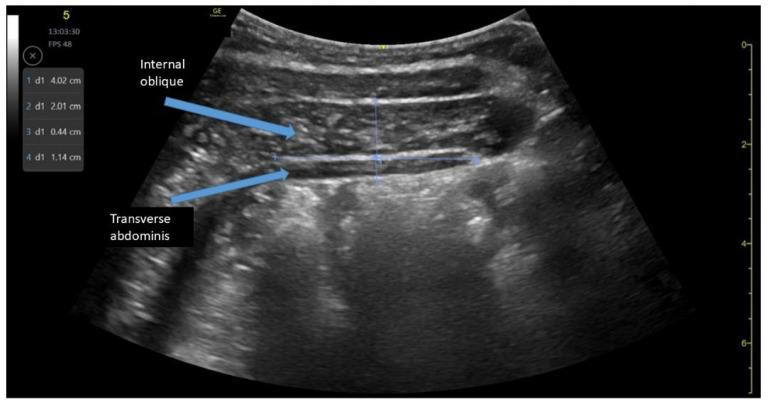
Ultrasound assessment of abdominal muscles at rest and muscle thickness measurements.

Abdominal muscles assessment was performed when the athlete was in the supine hook-lying position. The transducer was placed between the iliac crest and the ribs along the midaxillary line. To standardize the transducer’s position, the hyperechoic interface between the transverse abdominal (TrA) fascia and thoracolumbar fascia was aligned to the side of the ultrasound image. The angle of the transducer was then adjusted to optimize image visualization. The TrA and internal oblique muscles (IO) were measured at rest and during contraction (asking the athlete to perform the abdominal drawing-in maneuver). The image was frozen and saved at the end of expiration, following which the thickness measurements were obtained using the online caliper. Measurements were obtained on both right and left sides, at a set distance of 2 cm from the reference point (anterior medial border of TrA) (Figure 1) [14,15,16]. Two images of each muscle were captured, and the average of the two measurements was used for final analysis.

#### 2.3.2. Trunk Muscle Clinical Endurance Tests

Extension endurance and side bridge test results were included in this study. The side bridge exercise was performed with the athlete lying on their side with extended legs. The athlete was then required to lift the hips, supporting themselves on one elbow and their feet. The number of seconds held in this position was recorded. The extension test was performed while the athlete lay prone on the physio bed with the upper body off the bed and with legs fixed to the bed. The athletes were required to lift their upper body to form a straight line with the bed and maintain this position as long as possible. The time was recorded in seconds [17].

### 2.4. Data Analysis

The analysis was performed by the R Foundation for Statistical Computing version (4.3.1). The absolute values for TrA and IO muscle thickness were evaluated, as well as the percentage of change. The percentage of change was calculated as percentage thickness change from rest to the contracted position using the following formula: [(thickness contracted–thickness rest)/thickness rest] × 100 [18]. The ratio between the side bridge test (in seconds) and the trunk extension test (in seconds) was calculated for both the dominant and non-dominant sides (side flexion/extension) [19].

None of the continuous variables were normally distributed; therefore, we chose to use non-parametric tests. Continuous variables were reported as the median and interquartile range, and categorical variables were reported as proportions. The Wilcoxon signed rank sum test was used to evaluate the differences in all parameters between the dominant and non-dominant sides. The Wilcoxon two-sample test was used to evaluate the differences in all parameters across genders. Spearman correlation was calculated to explore associations between ultrasound-measured parameters and clinical tests. No multiple comparisons tests were performed, as the study design focused on a limited number of pre-specified hypotheses and group comparisons. The *p*-value for significance for all tests was 0.05.

The asymmetry across dominant and non-dominant sides for each measurement was determined using the method described by Rankin et al. (2006), [19] in which the absolute difference in values between the dominant and non-dominant sides was expressed as a percentage difference using the following formula: Asymmetry (%) = [(largest/smallest value × 100) − 100] [19].

According to physiotherapy-specific guidelines for effect size, for individual differences (Pearson’s r), small, medium, and large effect sizes were considered as 0.3, 0.5, and 0.6, respectively. For group differences (Cohen’s d or Hedges’ g), small, medium, and large effect sizes correspond to 0.1, 0.4, and 0.8, respectively [20].

This retrospective study included all athletes whose medical records were available from pre-season testing at the Ribstein Center for Sport Medicine and Research, Wingate Institute. The sample size was, therefore, determined by the number of records accessible during the data collection period.

## 3. Results

Data on 15 elite archery athletes (10 M and 5 F) were included in this study. The mean age was 17.2 (±2.7) years, and the mean height and weight were 170.56 (±11.01) cm and 71.8 (±20.8) kg, respectively. No athletes reported low back pain, yet two reported pain in their thoracic area. All athletes trained at least 7 units of archery training per week, each for 3 h, and completed another 2 sessions of strength and conditioning training per week.

### 3.1. Differences Between Sides

**Figure 2:**
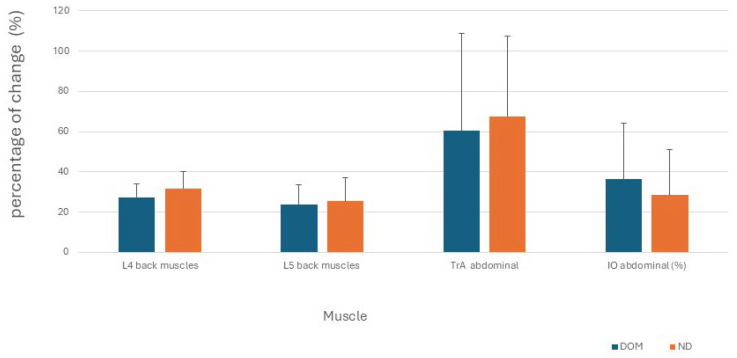
Percent thickness change from rest to contract position mean + standard deviation bars (following the formula [(thickness contracted–thickness rest)/thickness rest] × 100).

No differences were found between the dominant and non-dominant sides in all outcome measurements (absolute thickness and percentage of difference between rest and contraction positions) (*p* > 0.05, effect size small to medium). The results of the absolute variables are presented in Table 1, and the percentage of change values are in Figure 2.

### 3.2. Gender Differences

When examining gender differences in IO muscle thickness, males exhibited significantly greater values than females on the dominant side, both at rest and during contraction. At rest, the thickness was higher in males (median 0.91 [0.82;0.98]) compared with females (median 0.70 [0.63;0.76], *p* = 0.03, effect size r = 0.53, medium effect). During contraction, the difference remained significant (1.25 [1.07;1.37] vs. 0.90 [0.81;0.94], *p* = 0.02, effect size r = 0.6 (medium effect). No other differences between genders were found.

### 3.3. Symmetry and Asymmetry

The asymmetry of absolute size ranged from 4 to 47% for the IO and TrA and from 4 to 19% for the back muscles. Symmetry was better for the back muscles compared to the abdominal muscles, in which a large variation was noticed, as indicated by a large range and large standard deviation. Asymmetry was noticed in the side bridge clinical test, in which a large individual variation was noticed (Table 2). No differences in asymmetry were found between genders (*p* > 0.05, small effect size).

### 3.4. Correlation Between Muscle Morphology and Clinical Tests

The evaluation of the correlation between the clinical tests and the ultrasound parameters of muscle thickness and function revealed a strong correlation between the trunk non-dominant side bridge and the percentage of change in back muscle thickness (relationship between rest and contraction positions) at the L5 level (*p* < 0.05, r 0.6–0.75). No other correlations were found (Table 3).

## 4. Discussion

This study is the first to assess trunk muscles (abdominal and back muscles) in young archery athletes. Although archery is an asymmetrical sport, we found no differences in muscle thickness and function between the dominant and non-dominant sides. This aligns with other studies examining trunk muscle asymmetry in asymmetrical sporting activities [21,22,23]. Gill et al. [21] studied the asymmetry of abdominal muscles in collegiate single-sided rowers using ultrasound assessments. They found a significant difference in the TrA muscles, which were slightly thicker on the non-oarside. However, the authors noted that this difference was not clinically significant, and no other differences were observed in the absolute or relative thickness of the lateral abdominal muscles. No differences in trunk rotation strength between the dominant and non-dominant sides were observed in a study examining golf athletes using a Biodex isokinetic dynamometer [23]. In contrast, the total thickness of the abdominal muscles was greater on the non-dominant side than on the dominant side among fast bowlers, as assessed by ultrasound in a static position [24]. Zemková et al. [25] studied the peak and mean values of power during trunk rotation on the dominant and non-dominant sides in golfers, ice-hockey players, and tennis players, finding higher values on the dominant side. Hides et al. [26] examined, via MRI, abdominal, quadratus lumborum, psoas, and back muscles among elite cricketers, finding that the back muscle and quadratus lumborum were larger ipsilateral to the dominant arm and that the IO muscle was larger on the side contralateral to the dominant arm.

It is worth noting that even in the studies that found trunk muscle asymmetry, there is no consistency regarding which side is thicker or stronger, i.e., the dominant or the non-dominant side.

Our findings of trunk muscle symmetry might indicate that the essentially requirement for high trunk and upper extremity stability during archery results in the abdominal and back muscles being symmetrical in archery athletes, despite archery being an asymmetrical sport. Archers are trained to maintain a stable posture and minimize unnecessary trunk rotation. This may cause balanced isometrical muscle activity and help prevent excessive unilateral hypertrophy. This symmetry between sides is supported by the study by Hodges et al. [27], which examined abdominal muscle onset with EMG during arm movement. Their findings revealed that the onset of TrA occurred before the activation of the deltoid muscle, regardless of movement direction. They proposed that the TrA functions to control trunk stability independently of the direction of arm movement. The high demand for strong trunk muscles for stability was also found in a study by Azhar et al. [28], which examined abdominal and back muscles via EMG during different phases of shots in archers with different levels of training. Their findings suggest that elite archers had more activity of the muscles compared with the trained and beginner archers. However, they only measured one side, contralateral to the bow arm, without examining asymmetry between sides.

The trunk muscle symmetry found in our study might also be related to the young age of the sample. Thus, long-term adaptations to archery training may not have fully developed.

We found a small difference between genders, with the IO muscle thickness being larger in males compared to females on the dominant side, both at rest and during contraction. This difference was not observed for the back muscles. No differences between genders were observed regarding symmetry or differences between the dominant and non-dominant sides. Similar to our study, Gill et al. [21] examined the abdominal muscles in rowers and also found no difference in muscle asymmetry when accounting for gender. Other studies also indicate that differences in absolute muscle morphology are more pronounced in males than in females [29,30].

The current study revealed a greater degree of asymmetry in the abdominal muscles compared to the back muscles. This might be related to the demand needed from the muscles for trunk stability while standing, especially when holding the load of the bow. El Rich et al. [31] studied EMG activity in standing posture, holding weights with arms in front and on the side of the body. Their results showed that the trunk extensor muscle activity significantly increased when the load was held in front, yet not when the load was held on the sides, and that the abdominal muscles remained relatively silent. This is similar to the standing position in archery, where a load is held in front, suggesting that during standing, there is an increase in back muscle activity, which may work symmetrically to ensure trunk stability.

Our findings carry significant implications for both injury prevention and performance optimization in archery. Given the crucial role of trunk muscles in maintaining trunk stability for shooting accuracy, core training programs should be performed and focus on improving endurance in both the abdominal and back muscles. Additionally, pre-season screening should be conducted to assess muscle strength and identify muscle imbalances. This may help identify athletes at higher risk of asymmetrical patterns, potentially predisposing them to overuse injuries.

### 4.1. Study Limitations and Future Studies

This study has several limitations. The sample size was relatively small, which may limit the applicability of our findings to a wider population of archers. This also addresses the differences between genders, as our sample included only five females, and the observed difference should be further examined. Second, ultrasound measurements were taken at rest and during specific contraction tasks, but dynamic assessments of muscle activation during drawing and releasing an arrow were not included. Another limitation is the absence of a control group. Future studies using electromyography and a motion capture analysis system during actual archery sequences could provide deeper insights into joint movements and muscle activation patterns. Additionally, studies on older or more experienced archers should be performed, as they might reveal greater muscle imbalances due to cumulative exposure to asymmetrical loading. A comparison with other symmetrical and asymmetrical sports should be explored.

### 4.2. Clinical Recommendations

Pre-season screening of young competitive archers should be performed and include assessment of trunk muscle symmetry. Although overall symmetry was noted, sex-related differences and individual variability in abdominal muscle thickness may require targeted core stability training. Early detection of such asymmetries could help optimize performance and reduce the risk of overuse injuries in this population. Routine core muscle exercises are recommended, as these muscles play a key role in maintaining appropriate trunk stability during archery and may contribute to improved performance.

## 5. Conclusions

Despite the asymmetrical functional demands of sport archery, young athletes displayed trunk muscle symmetry, particularly in their back muscles. While some variability in abdominal muscle asymmetry was observed, these differences were not statistically significant. Experienced archery athletes should be studied to assess the impact of prolonged asymmetrical loads on trunk muscle symmetry.

## Figures and Tables

**Table 1:** Results of ultrasound measurements and trunk muscle clinical tests in dominant and non-dominant sides.

	DOM (N = 15) Median (Q1, Q3)	ND (N = 15) Median (Q1, Q3)	Difference (N = 15)	*p* Value	ES
Ultrasound measurements					
AVE_Back_Rest_L4 cm	3.35 (2.839, 3.671)	3.14 (2.755, 3.415)	−0.147 (−0.312, 0.080)	0.07	0.48
AVE_Back_Rest_L5 cm	3.070 (2.748, 3.459)	3.033 (2.610, 3.287)	−0.005 (−0.205, 0.172)	0.87	0.04
AVE_Back_Cont_L4 cm	4.305 (3.550, 4.554)	4.105 (3.857, 4.412)	−0.080 (−0.211, 0.100)	0.36	0.24
AVE_Back_Cont_L5 cm	3.833 (3.282, 4.245)	3.763 (3.460, 4.171)	−0.057 (−0.220, 0.110)	0.65	0.13
AVE_TrA_Rest cm	0.465 (0.325, 0.518)	0.420 (0.385, 0.565)	0.020 (−0.063, 0.087)	0.73	0.09
AVE_IO_Rest cm	0.840 (0.705, 0.945)	0.885 (0.692, 1.075)	0.050 (−0.052, 0.145)	0.19	0.34
AVE_TrA_Cont cm	0.700 (0.545, 0.830)	0.760 (0.627, 0.857)	0.030 (−0.045, 0.097)	0.65	0.12
AVE_IO_Cont cm	1.070 (0.917, 1.337)	1.025 (0.920, 1.400)	−0.065 (−0.125, 0.210)	0.75	0.08
Clinical tests					
Trunk_SF__sec	92.000 (62.000, 107.000)	92.000 (59.750, 126.750)	6.000 (0.000, 25.000)	0.34	0.29
Side_bridge__Ext_Ratio	0.808 (0.523, 0.899)	0.761 (0.523, 0.989)	0.107 (0.000, 0.210)	0.30	0.31

DOM—dominant side, ND—non dominant side, ES—effect size.

**Table 2:** The degree of asymmetry of the absolute size of the trunk muscles shown as the percentage difference between the sides.

	Interside Difference (%)	SD
AVE_Back_Rest_L4	7.9	5.15
AVE_Back_Cont_L4	5.1	4.58
AVE_Back_Rest_L5	7.5	4.15
AVE_Back_Cont_L5	5.9	4.70
AVE_TrA_Rest	18.8	12.02
AVE_TrA_Cont	16.2	13.05
AVE_IO_Rest	15.4	12.62
AVE_IO_Cont	16.5	8.69
Trunk_SF	44.8	31.02

TrA—transverse abdominis muscle, IO—internal oblique muscle, Cont—contract, SF—side flexion clinical test.

**Table 3:** Correlation between clinical tests and ultrasound examination parameters of back muscles.

	Trunk Extension R (*p* Value)	Trunk Dominant Side Bridge R (*p* Value)	Trunk Non-Dominant Side Bridge R (*p* Value)
DOM_L4_percent	0.139 (0.63)	0.265 (0.40)	0.239 (0.43)
DOM_L5_percent	0.117 (0.69)	0.209 (0.51)	0.660 (**0.01**)
ND_L4_percent	0.214 (0.46)	0.349 (0.26)	0.514 (0.07)
ND_L5_percent	0.278 (0.33)	0.454 (0.13)	0.751 (**0.01**)
Percent_DOM_TrA	−0.001 (0.99)	0.335 (0.26)	0.173 (0.55)
Percent_DOM_IO	0.030 (0.91)	0.450 (0.12)	0.332 (0.24)
Percent_ND_TrA	−0.168 (0.54)	0.065 (0.83)	0.055 (0.85)
Percent_ND_IO	0.127 (0.65)	0.197 (0.51)	0.050 (0.86)

Bold = significant < 0.05. TrA—transverse abdominis muscle, IO—internal oblique muscle, DOM—dominant, ND—non dominant.

## Data Availability

The data that support the findings of this study are available from the corresponding author upon reasonable request.
